# Estimating the economic impact of COVID-19 disruption on access to sexual and reproductive health and rights in Eastern and Southern Africa

**DOI:** 10.3389/fpubh.2023.1144150

**Published:** 2023-06-22

**Authors:** Hillary Kipchumba Kipruto, Humphrey Cyprian Karamagi, Solyana Ngusbrhan Kidane, Daniel Mwai, David Njuguna, Benson Droti, Wangui Muthigani, Easter Olwanda, Elvis Kirui, Ayotunde Adenola Adegboyega, Amaka Pamela Onyiah, Juliet Nabyonga-Orem

**Affiliations:** ^1^Health Information System, Universal Health Coverage Life Course, WHO Regional Office for Africa, Harare, Zimbabwe; ^2^Data, Analytics and Knowledge Management, WHO Regional Office for Africa, Brazzaville, Democratic Republic of Congo; ^3^Health Economics Unit, University of Nairobi, Nairobi, Kenya; ^4^Ministry of Health, Nairobi, Kenya; ^5^Reproductive Maternal New-born and Child and Adolescent Health, Universal Health Coverage Life Course, WHO Regional Office for Africa, Harare, Zimbabwe; ^6^Reproductive Maternal Health and Ageing, Universal Health Coverage Life Course, WHO Regional Office for Africa, Brazzaville, Democratic Republic of Congo; ^7^Health Financing, Universal Health Coverage Life Course WHO Regional Office for Africa Harare, Harare, Zimbabwe

**Keywords:** COVID-19, Disability Adjusted Life Years, disruption, health system, deliveries, Eastern and Southern Africa, antenatal visits

## Abstract

**Background:**

The Coronavirus disease 2019 (COVID-19) resulted in the disruption of Sexual and Reproductive Health Rights (SRHR) services in the Eastern and Southern Africa region. To date, studies estimating the impact of COVID-19 disruptions have mainly focused on SRHR services without estimating the economic implication.

**Method:**

We used national service coverage data on the effectiveness of interventions from the lives saved tool (LiST), a mathematical modeling tool that estimates the effects of service coverage change in mortality. We computed years lost due to COVID-19 disruption on SRHR using life expectancy at birth, number of years of life lost due to child mortality, and life expectancy at average maternal death. We calculated the economic value of the lives saved, using the values of statistical life year for each of the countries, comparing 2019 (pre-COVID-19) to 2020 (COVID-19 era).

**Findings:**

The total life-years lost were 1,335,663, with 1,056,174 life-years lost attributed to child mortality and 279,249 linked to maternal mortalities, with high case-fatality rates in the Democratic Republic of Congo, Burundi, and Tanzania. The findings show COVID-19 disruptions on SRHR services between 2019 and 2020 resulted in US$ 3.6 billion losses, with the highest losses in Angola (USD 777 million), South Africa (USD 539 million), and Democratic Republic of Congo (USD 361 million).

**Conclusion:**

The monetized value of disability adjusted life years can be used as evidence for advocacy, increased investment, and appropriate mitigation strategies. Countries should strengthen their health systems functionality, incorporating and transforming lessons learned from shock events.

## Highlights

- The COVID-19 disruption on sexual and reproductive health rights services across the Eastern and Southern Africa region resulted in 1,056,174 life-years lost attributed to child mortality and 279, 249 life-years lost linked to maternal mortalities.
- COVID-19 disruptions in sexual and reproductive health rights services caused a 3.6 USD billion loss in Eastern and Southern Africa countries in 2020.
- Countries need to strengthen their health systems functionality, incorporating and transforming lessons learned from shock events and avert health services disruption.

## Introduction

The Coronavirus disease 2019 (COVID-19) pandemic declared in March 2020 caused unprecedented disruptions to all spheres of life, and led to uncertainty and apprehension globally (1). COVID-19 has caused extensive changes to healthcare systems globally, including large reductions in services, particularly in settings impacted significantly by the pandemic (2). As a new disease, it has also provoked the reluctance to screen and access treatment (3). Public health measures were deployed as mitigation, including lockdowns, infection and prevention control measures, and vaccination amongst others, which have had positive and negative impacts (4). Some of the undesired consequences include population fatigue to compliance with public health measures, reduced or delayed healthcare utilization, and varied socio-economic impact on society. While the first cases in Eastern and Southern Africa were identified later than other parts of the world, drastic lockdown measures were adopted to contain the spread of the virus.

Sexual and reproductive health refers to “the state of physical, social, mental, and emotional well-being related to sexuality and reproductive health, and not merely the absence of disease, dysfunction or infirmity” (5). Although sexual and reproductive health and rights (SRHR) is not limited to youth and adolescent, the Eastern and Southern Africa sub-region has more than 165 million young people aged between 10 and 24 years, and this population is anticipated to rise to 263 million by 2050. However, young people experience challenges with sexual and reproductive health (6). The Eastern and Southern Africa region has one of the world’s highest rates of HIV infection, with 625,000 new infections occurring annually (7).

Conversely, the COVID-19 pandemic, and particularly the mitigation policies, had an adverse effect on sexual and reproductive health services and threatened to reverse the gains made toward the Sustainable Development Goals. Reduced or delayed access and healthcare utilization of sexual and reproductive health services during the pandemic can have detrimental health consequences that will likely create new challenges for women, young girls, and the entire healthcare system (8). The economic consequences are also likely to deepen poverty including the feminization of poverty, (9) pre-dispose women and young girls to disproportionate social and economic risk, which may reverse advances made to achieve gender equality (10).

Simulation studies have modeled the impact of COVID-19-related disruptions in access to essential SRHR services. According to estimates by Church et al., the service disruptions at Marie Stopes International-affiliated health facilities across 37 countries imply that the pandemic could result in additional 5,000 pregnancy-related deaths, 1.3 million unintended pregnancies and 1.2 million unsafe abortions (11). Without mitigation strategies, they projected between 13 million and 51 million women who otherwise would have used modern contraceptives will be unable to (12). Similarly, estimates by the United Nations Population Fund suggest that up to 51 million women in low and middle-income countries might not be able to access modern contraceptives due to health service disruption, resulting in close to 15 million unintended pregnancies (13).

In the Sub-Saharan Africa, supply disruption of the antiretroviral therapy was predicted to cause greater than 500,000 additional deaths, between 2020 and 2021 (14). The Guttmacher institute estimated a potential annual impact of a 10% decline in the use of SRHR services, resulting from COVID-19–related disruptions, in 132 low- and middle-income countries. In 1 year, they projected 49 million more women would have an unmet need for contraception, 15 million more unintended pregnancies, 168,000 more new-borns deaths, and 28,000 more maternal deaths. Besides, inefficient policies and structural barriers may contribute to millions of people losing access to essential sexual and reproductive health services (15).

The studies above have outlined the subsequent impact of the disruptions on SRHR, which is viewed as the difference between the actual coverage during the pandemic and the anticipated service coverage in the absence of COVID-19. The magnitude of the impact largely depends on how baselines and disruptions are conceptualized and measured (16). Others have utilized mortality rates of COVID-19, which are a critical measure of a disease’s impact. This can aid policymakers and healthcare providers to understand the extent of the outbreak and provide insight into the impact of the disease, across geographic areas and sociodemographic variables. The COVID-19 mortality rates can also highlight the past and future trends of the pandemic. However, they do not offer sufficient information on the number of Years of Life Lost (YLL) due to the disease.

Impacts of the pandemic may be direct or indirect, as a result of change in the healthcare system or public health measures used to mitigate the pandemic, such as lockdown measures. Previous studies evaluating COVID-19 control measures have primarily focused on epidemiological outcomes. These studies have estimated the mortality rates and hospitalization rates of COVID-19 and how it might vary across sub-populations (17). However, the reported numbers of hospitalizations and deaths provide limited insight into the state of the pandemic. Besides, most studies report the count of deaths directly attributable to the COVID-19 but do not cover indirect deaths resulting from limited or no access to public health measures such as SRHR services. To date, studies estimating economic impact of SRHR services disruption, during the COVID-19 pandemic, in the Eastern and Southern Africa region have not yet been carried out. This analysis provides a comprehensive account of the Disability Adjusted Life Years (DALYs) and YLL following disruptions of SRHR services during the COVID-19 pandemic, from March 2020 to March 2021. It also provides insights into decision-making trade-offs, in the context of the COVID-19 mitigation, that apply to the Eastern and Southern Africa region.

## Methods

We used national data on service coverage jointly with information on the effectiveness of interventions from the lives saved tool (LiST) (18), a mathematical modeling tool that estimates the effects of service coverage change on mortality in low and middle-income countries. The LiST tool has been used in several studies to quantify the number of lives saved because of SRHR health programs.

In this study, LiST was used to estimate the health impact of scaling down SRHR services and interventions based on the estimated disruption level per country. The impact of COVID-19 disruption was quantifiable in terms of the disease burdens for each country. Each LiST projection calculated an annual number of deaths based on SRHR intervention coverage changes relative to baseline, which is the pre-COVID-19 values for the year 2019. The difference in the number of deaths calculated each year between the paired LiST projections was the annual number of lives lost for each country, expressed as crude numbers per specific population of the countries. The values are also expressed per 1,000 for child lives lost and per 100,000 for maternal lives lost, per country.

The increase in disease burden estimated with DALYs was generated. DALY is calculated as a sum of years lost because of premature mortality, and the years lived with disability due to preventable condition or disease (19). YLL is the product of the number of lives lost, and factors the standard life expectancy at the age at which death occurs (19). In this analysis, we calculated the YLL by multiplying the number of lives lost following the SRHR intervention scale down by the life expectancy. The standard life expectancy for the age when child mortality occurs and the life expectancy at average maternal death was used, to estimate the total YLL. The standard life expectancy used for child mortality was 2.5 years less than the life expectancy at birth. The maternal life expectancy was calculated based on the average childbearing age, for each country.

Monetized DALYs is based on a constant value per statistical life year derived from a value per statistical life estimate. There is uncertainty in value per statistical life, as it does not represent the inherent value of life but condenses actual and stated trade-offs people make in choosing between money and small changes in mortal risk (20). The value per statistical life year is provided by the residue of life expectancy and age of death.

The life years lost due to COVID-19 disruption on SRHR was computed using the number of lives lost generated by LiST, life expectancy at birth, number of YLL due to child mortality, and life expectancy at average maternal death (21). We calculated the economic value of the lives saved using the values of statistical life year (22) for each country in the Eastern and Southern Africa region. The data used in this analysis are annual estimates, and the reference year is March 2019– March 2020 (pre-COVID-19) and March 2020–March 2021 (COVID-19 era). Data management and analysis were conducted using Microsoft Excel and STATA 14.

Productivity benefits refer to the economic benefits that a country will enjoy as a result of a healthier and more productive population. These benefits have been estimated from the number of lives saved due to the scaling down of various SRHR interventions across countries following the COVID-19 interruptions.

Productivity benefit was calculated as follows:

$$\begin{array}{c} Productivitybenefits=Presentvalueof\;a\;statisticallife\\ year\;\left(USD\right)\times Numberoflivessaved \end{array}$$

**Figure 1:**
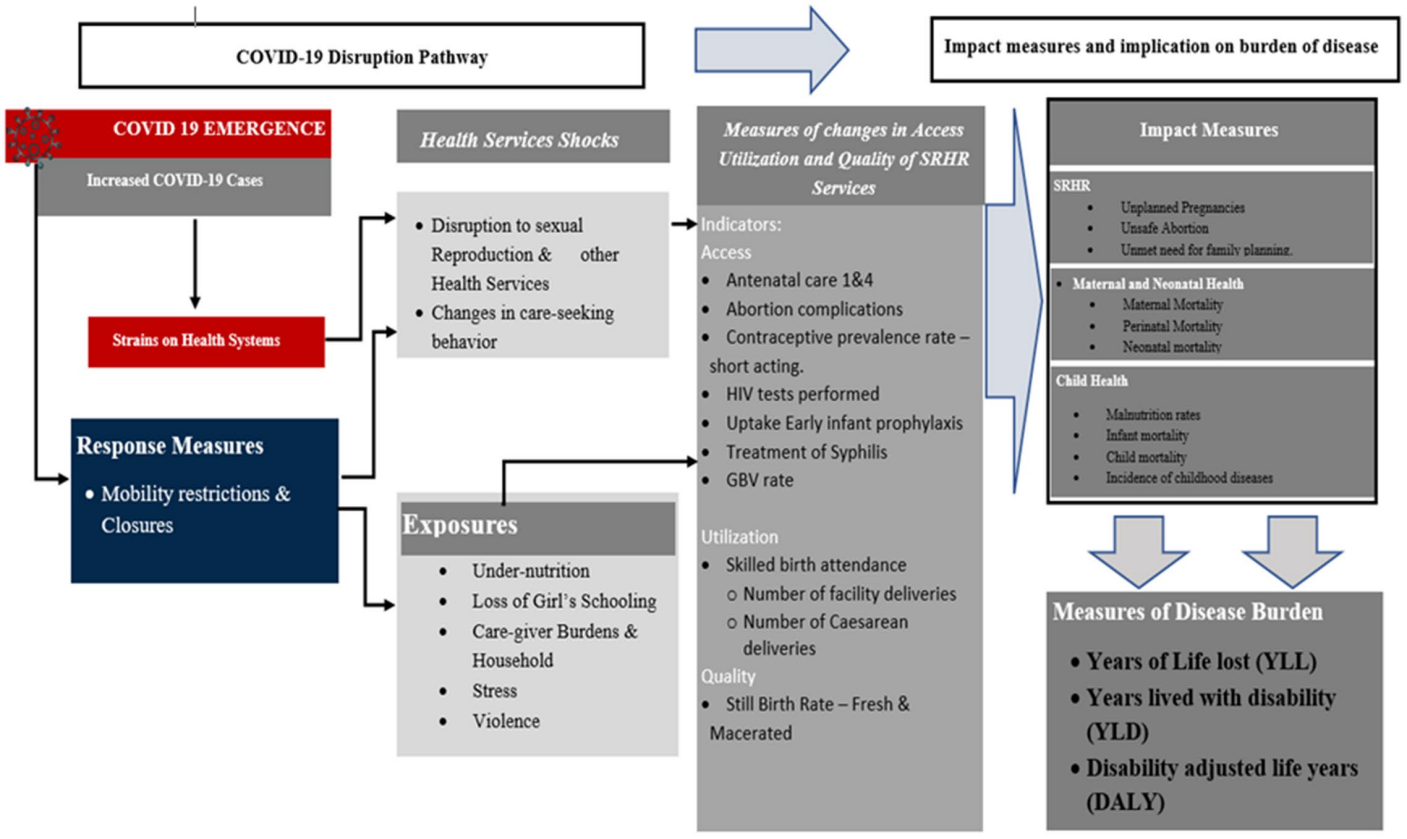
Conceptual analysis pathway, from the emergence of COVID-19 to the impact on disease burden.

Overall, the conceptual pathway for the analysis, from the emergence of COVID-19, through the disruption of SRHR services, to the impact on disease burden is outlined in Figure 1.

## Findings

**Figure 2:**
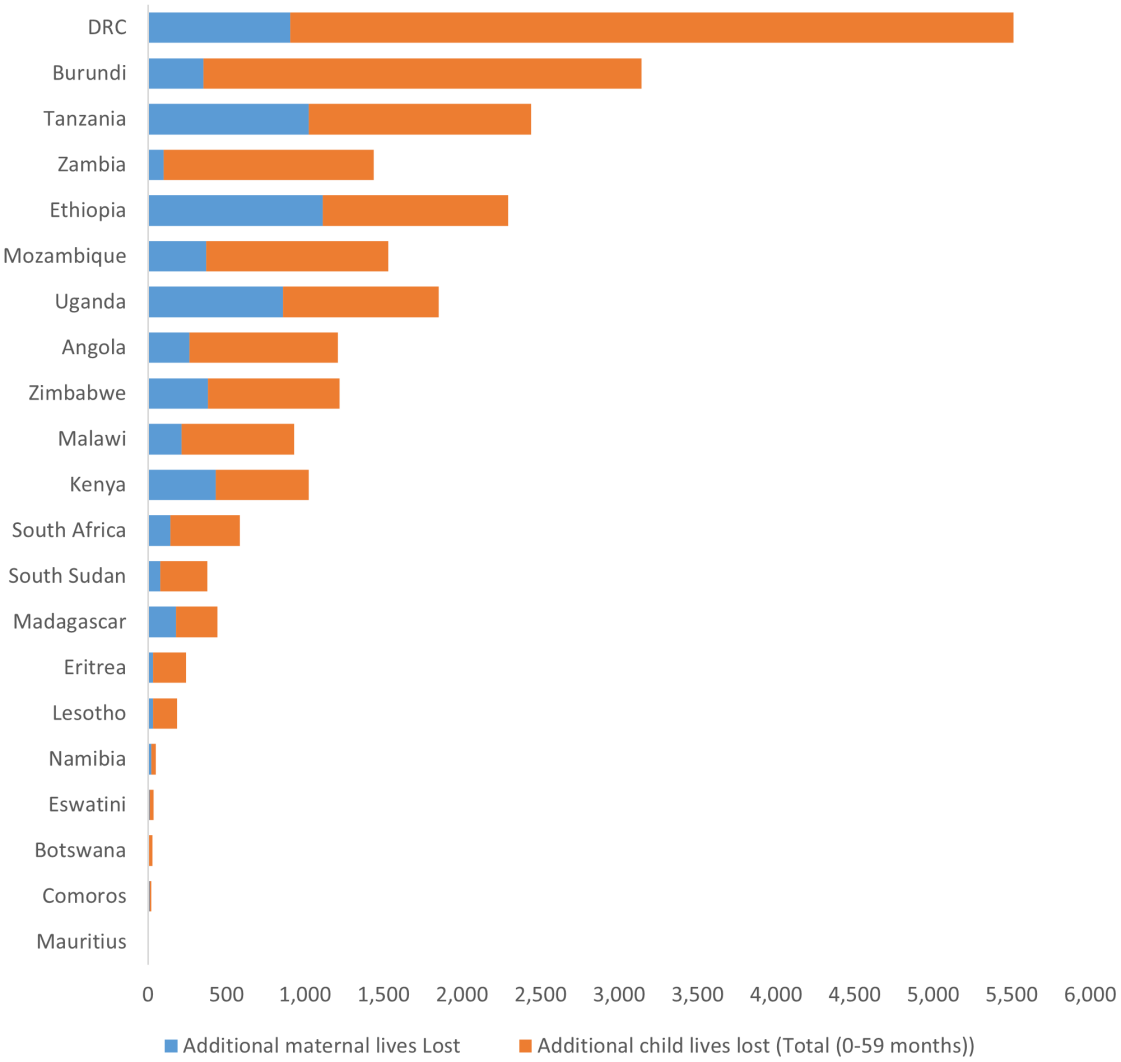
Additional lives (maternal and child) lost in the Eastern and Southern Africa region, 2020–21.

The findings of this study show that Mauritius, Botswana, Eswatini, Comoros, Namibia, Lesotho, and Eritrea reported the lowest maternal mortalities (ranging between 1 and 32), while Uganda, Democratic Republic of Congo, Tanzania, and Ethiopia reported the highest maternal mortalities (ranging between 860 and 1,113). The findings also show that Mauritius, Comoros, Botswana, Eswatini, and Namibia lost the lowest number of children (ranging between 2 and 28) while Uganda, Mozambique, Ethiopia, Zambia, Tanzania, Burundi, and Democratic Republic of Congo lost the highest number of children (ranging between 990 and 4,606). See Figure 2.

**Figure 3:**
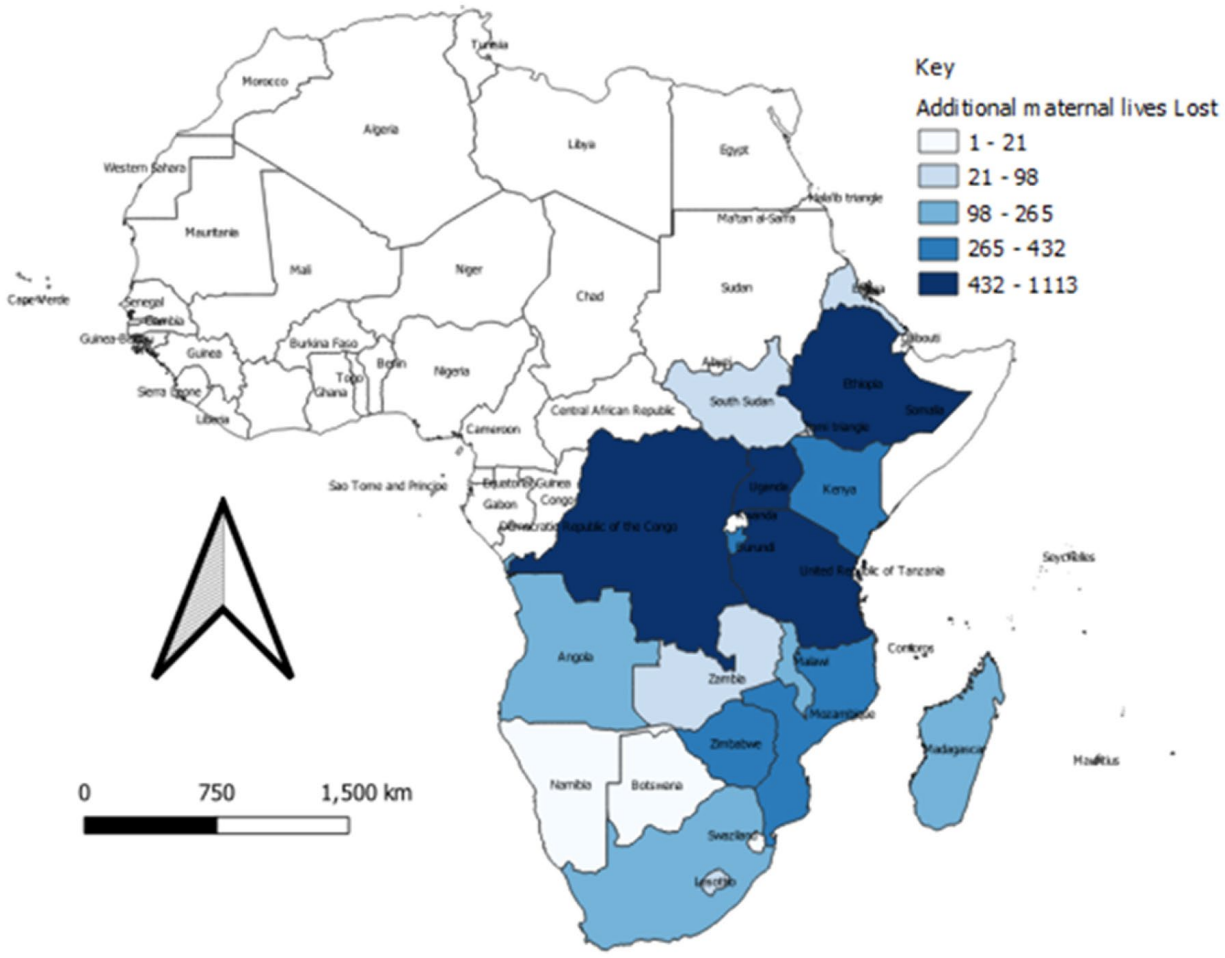
Spatial distribution of additional maternal mortality during 2020–21 across the Eastern and Southern Africa region.

**Figure 4:**
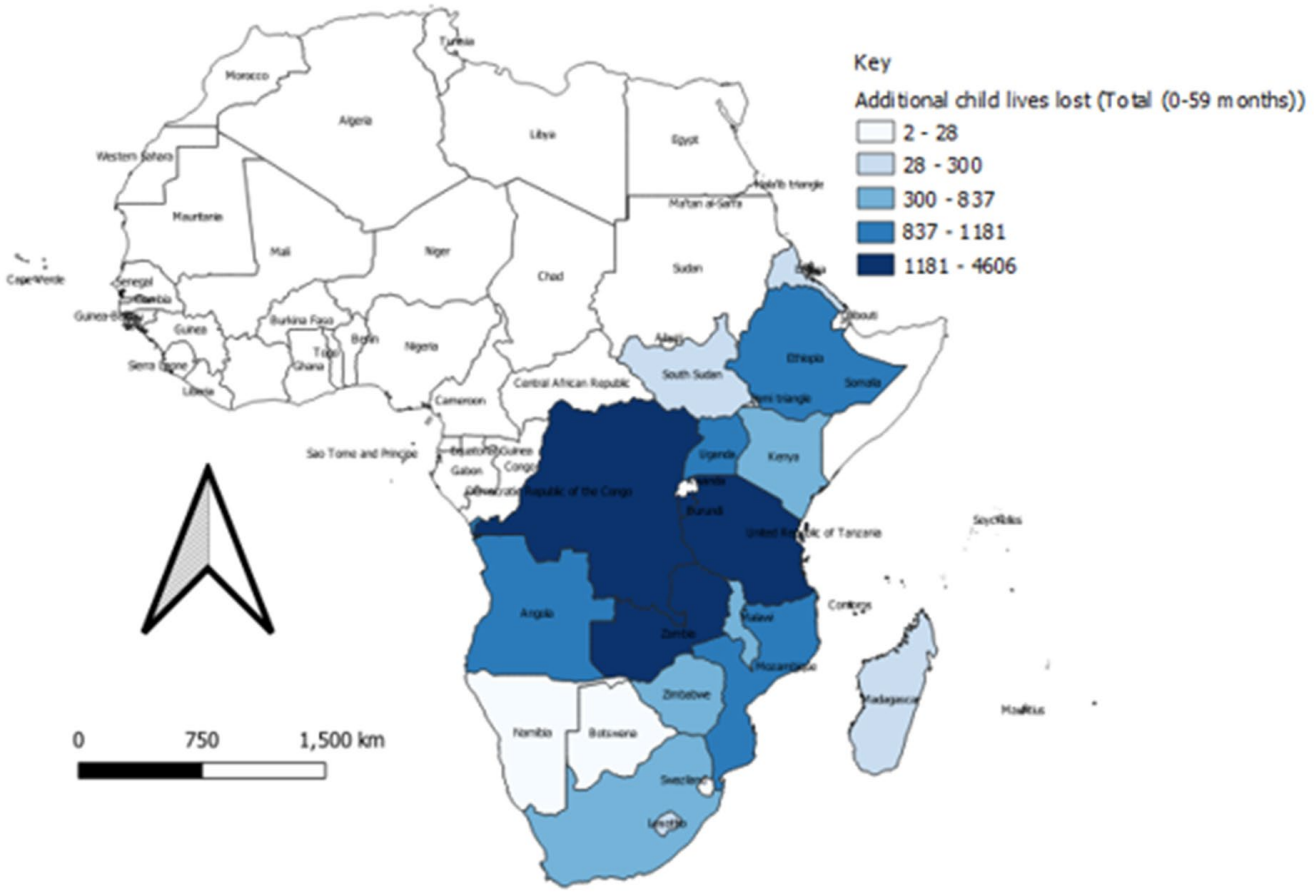
Spatial distribution of child mortality during 2020–21 in the Eastern and Southern Africa region.

The impact of COVID-19 disruption of SRHR intervention varied across the countries in the and Southern Africa region. Figures 3, 4 show the spatial distribution of lives lost from maternal and child mortalities.

### Number of life years lost

**Figure 5:**
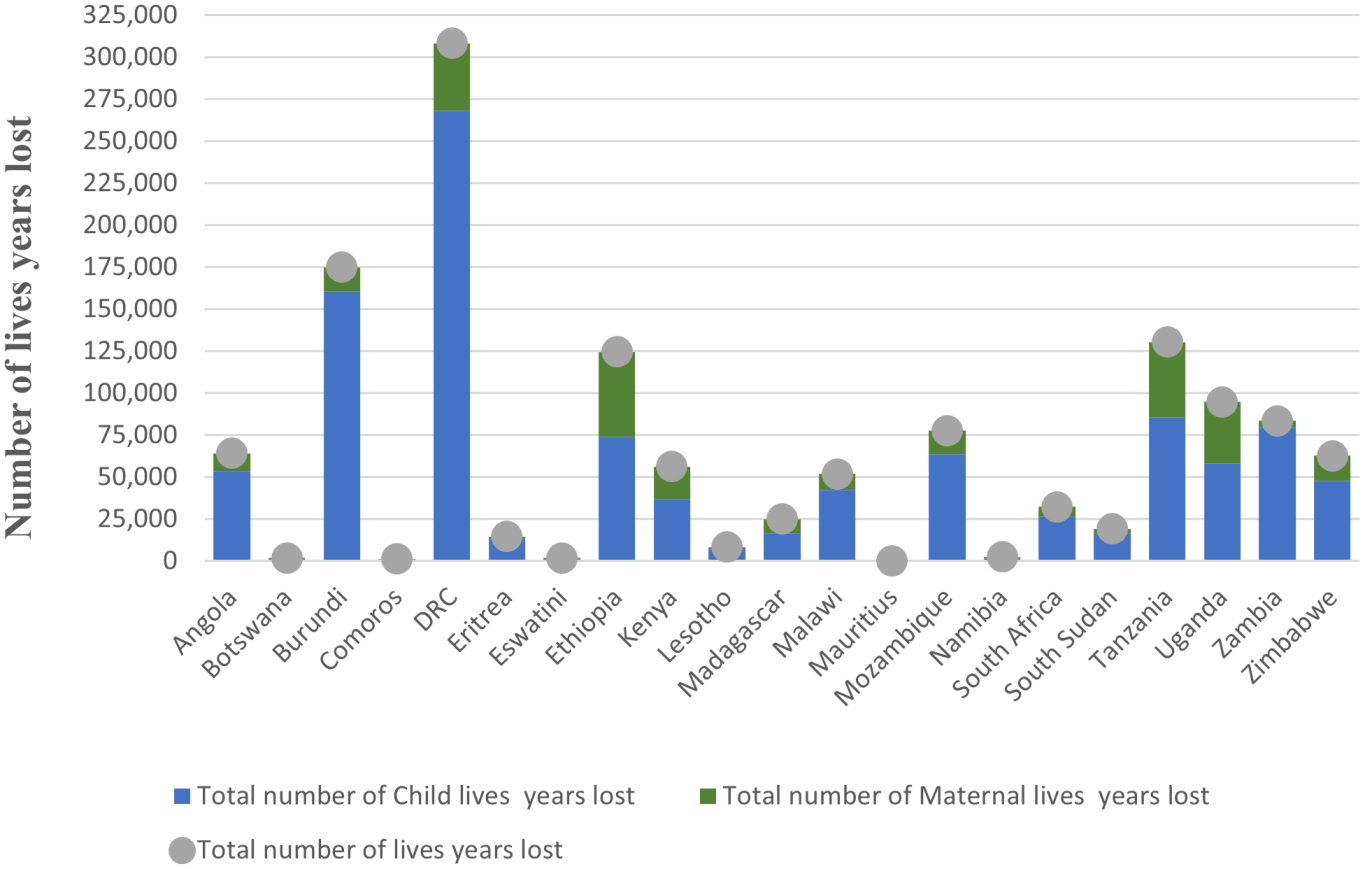
Number of life years lost by country in the Eastern and Southern Africa region, 2020–21.

The COVID-19 disruption of SRHR services across the Eastern and Southern Africa region resulted in significant loss of life years. Across the countries, the total life-years lost were 1,335,663. A total of 1,056,174 life-years lost were attributed to child mortality while 279,249 life-years lost were linked to maternal mortalities. Details of the breakdown are reflected in Table 1 and Figure 5.

**Table 1:** Number of years of life lost by country, 2020–21.

Country	Total number of child life years lost	Total number of maternal life years lost	Total number of lives years lost
Mauritius	144	54	198
Comoros	732	446	1,177
Eswatini	1,427	318	1,745
Botswana	1,556	237	1,793
Namibia	1,669	855	2,524
Lesotho	7,426	955	8,381
Eritrea	13,117	1,410	14,527
South Sudan	16,307	2,835	19,141
Madagascar	16,705	8,195	24,900
South Africa	26,646	5,661	32,307
Malawi	42,509	9,256	51,765
Kenya	36,818	19,439	56,257
Zimbabwe	47,737	14,950	62,687
Angola	53,769	10,430	64,198
Mozambique	63,568	14,172	77,740
Zambia	79,378	4,169	83,546
Uganda	58,284	36,527	94,811
Ethiopia	73,869	50,584	124,453
Tanzania	85,825	44,512	130,337
Burundi	160,711	14,194	174,904
DRC	267,977	40,292	308,269
Total	1,056,174	279,489	1,335,663

The spatial distribution of total and additional number of child life years lost, as well as maternal life years lost for specific countries, is presented in Table 2. The additional lives lost is expressed in total number and as a rate (per population). The highest rate of additional child lives lost, and additional maternal deaths was reported in Burundi, 1.37 per 1,000 and 13.02 per 100,000, respectively.

**Table 2:** Additional number and rate per population of child and maternal years life lost vs. total, East and Southern Africa region, 2020–21.

	Additional child lives lost [Total (0–59 months)]	Additional child deaths per 1,000	Total number of child lives years lost	Additional maternal lives lost	Additional maternal deaths per 100,000	Total number of maternal lives years lost
Angola	945	0.17	53,769	265	3.61	10,430
Botswana	24	0.09	1,556	5	0.8	237
Burundi	2,789	1.37	160,711	352	13.02	14,194
Comoros	12	0.1	732	10	4.78	446
DRC	4,606	0.3	267,977	904	4.69	40,292
Eritrea	211	0.42	13,117	32	3.93	1,410
Eswatini	27	0.19	1,427	9	3	318
Ethiopia	1,181	0.07	73,869	1,113	4.04	50,584
Kenya	591	0.08	36,818	432	3.18	19,439
Lesotho	153	0.61	7,426	32	5.81	955
Madagascar	265	0.07	16,705	178	2.69	8,195
Malawi	715	0.25	42,509	215	4.78	9,256
Mauritius	2	0.03	144	1	0.3	54
Mozambique	1,162	0.23	63,568	369	5.09	14,172
Namibia	28	0.08	1,669	21	3.25	855
South Africa	443	0.08	26,646	141	0.88	5,661
South Sudan	300	0.18	16,307	77	2.96	2,835
Tanzania	1,416	0.15	85,825	1,023	7.57	44,512
Uganda	990	0.13	58,284	860	8.15	36,527
Zambia	1,340	0.46	79,378	98	2.26	4,169
Zimbabwe	837	0.41	47,737	383	10.48	14,950

The percentage of additional child lives lost in Eastern and Southern Africa ranged between 0.6% (Madagascar) to 10% (Burundi), whereas the additional maternal lives lost ranged from 0.5% (Zambia) to 12.8% (Burundi), as depicted in Table 3.

**Table 3:** Additional child and maternal lives lost in %, East and Southern Africa region, 2020–21.

	Total under 5 deaths	Additional under 5 death (crude)	Additional under 5 death covid (%)	Total maternal deaths	Additional maternal deaths (crude)	Additional maternal deaths (%)
Angola	95,875	945	1.0%	4,683	265	5.70%
Botswana	2,209	24	1.1%	284	5	1.80%
Burundi	27,917	2,789	10.0%	2,744	352	12.80%
Comoros	1,713	12	0.7%	119	10	8.40%
DRC	304,334	4,606	1.5%	19,412	904	4.70%
Eritrea	4,306	211	4.9%	1,545	32	2.10%
Eswatini	1,434	27	1.9%	277	9	3.20%
Ethiopia	185,999	1,181	0.6%	18,605	1,113	6.00%
Kenya	69,308	591	0.9%	10,451	432	4.10%
Lesotho	5,137	153	3.0%	907	32	3.50%
Madagascar	44,899	265	0.6%	6,369	178	2.80%
Malawi	27,327	715	2.6%	3,411	215	6.30%
Mauritius	231	2	0.9%	195	1	0.50%
Mozambique	82,509	1,162	1.4%	5,870	369	6.30%
Namibia	2,913	28	1.0%	326	21	6.40%
South Africa	39,633	443	1.1%	4,852	141	2.90%
South Sudan	36,339	300	0.8%	326	21	6.4%
Tanzania	105,811	1,416	1.3%	284	5	1.8%
Uganda	76,833	990	1.3%	277	9	3.2%
Zambia	41,228	1,340	3.3%	195	1	0.5%
Zimbabwe	25,962	837	3.2%	119	10	8.4%

### Monetization of health outcomes

**Figure 6:**
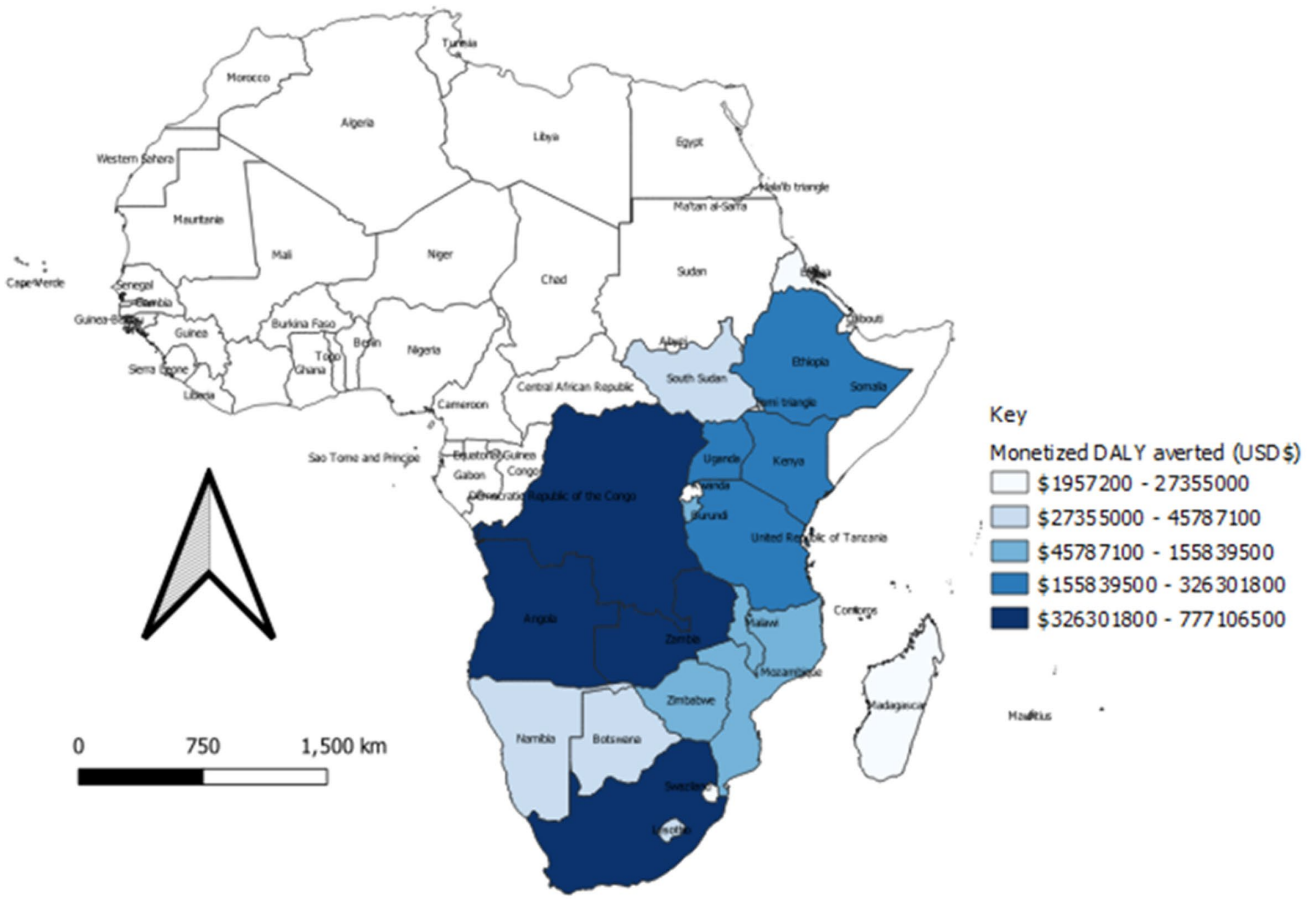
Monetized DALYs averted (USD$) in the Eastern and Southern Africa region, 2020–21.

The results show that COVID-19 disruptions in SRHR services resulted in 3.6 USD billion loss in Eastern and Southern Africa countries in 2020. The countries that experienced the greatest economic loss were Democratic Republic of Congo, South Africa, and Angola, which lost 361 USD million, 539 USD million, and 777 USD million, respectively. On the other hand, Eswatini, Comoros, and Mauritius had the lowest losses, standing at 2 million USD, 2.5 million USD, and 4.5 USD million, respectively. Table 4 and Figure 6 shows the number of years lost and the resulting monetized DALYs not averted due to the COVID-19 disruption of the SRHR services, across the counties in Eastern and Southern Africa.

**Table 4:** Total number of lives years lost vs. monetized DALYs averted (USD$).

Country	Total number of lives years lost	Monetized DALYs (USD$)
Eswatini	1,745	1,957,233
Comoros	1,177	2,485,647
Mauritius	198	4,482,198
Eritrea	14,527	13,950,654
Madagascar	24,900	27,355,009
Botswana	1,793	29,577,844
Lesotho	8,381	36,126,976
Namibia	2,524	36,290,296
South Sudan	19,141	45,787,121
Malawi	51,765	48,462,331
Burundi	174,904	130,909,817
Mozambique	77,740	138,613,065
Zimbabwe	62,687	155,839,471
Uganda	94,811	185,379,988
Ethiopia	124,453	195,151,070
Kenya	56,257	200,552,882
Tanzania	130,337	326,301,830
Zambia	83,546	346,434,533
DRC	308,269	360,697,392
South Africa	32,307	539,404,280
Angola	64,198	777,106,499
ESA – Total	1,335,663	3,602,866,137

In line with the extent of COVID-19 disruption on SRHR services per country, Figure 6 presents the special distribution of the economic losses per country. The value of benefits lost on account of COVID-19 is further displayed in the maps that follow.

## Discussion

The findings of the study show that the COVID-19 pandemic disruptions triggered maternal and child mortality crisis, thereby posing additional challenges to the population’s health. Across the countries, there was additional child lives lost (6,520 in total), varying from 0.6 to 10%. For maternal deaths, there was additional 18,037 lives lost, which ranged from 0.5 to 12.8% across countries. This finding aligns with that of Soma-Pillay et al. (23) who the impact of COVID-19 first wave monitored in South Africa, specifically on the use of maternal and reproductive health services as well as maternal mortality. Since the start of lockdown, they reported an increase of 30% (132) in maternal deaths since in 2020, in contrast to the same duration in 2019. The analysis noted decline in the use of reproductive health services, and increased pressure on rural provinces, as the result of pregnant women relocating to their homes (23).

The scale of additional mortalities experienced following the COVID-19 SRHR service disruption is devastating, considering that every death is a tragedy. The finding suggests that lockdown protocols could increase the country’s maternal and child mortality rate. These findings reveal three important deductions. First, policies and laws that obstruct access to SRHR services should be revised. Second, governments must adopt a patient-centered and human rights-based approach. Third, governments must also adapt policies and innovative service-delivery modalities, such as telemedicine, to guarantee access to SRHR services during shock events (24).

The COVID-19 disruptions on SRHR services have contributed to a sharp decline in life expectancy, as demonstrated in the total and additional YLL. This finding is consistent with Islam et al. (25) who reported more than 28 million excess YLL in 31 upper-middle- and high-income countries, in 2020. The excess YLL associated with the COVID-19 pandemic in 2020 were five times higher than those associated with the seasonal influenza epidemic in 2015. Both total and additional deaths were unevenly distributed across Eastern and Southern Africa countries, exacerbating pre-pandemic inequalities. Moreover, increased mortality rates among children contributed the most to life-expectancy declines across countries. The heightened YLL among children indicates premature mortality and calls for further investigations into the specific causes of child mortality. Overall, this finding underscores the importance of successful infection prevention control and mitigation policies, including targeted and population-based public health policy interventions. It also highlights the need for urgent efforts to ensure the continuity of SRHR services to avert additional YLL during the COVID-19 pandemic. The Eastern and Southern Africa countries should prioritize strengthened supply chain systems and promote equitable access to SRHR services. Moreover, a comprehensive pandemic preparedness aimed at more inherent resilient health systems could be key to tackling the impact of future pandemics.

The Eastern and Southern Africa region would salvage DALYs worth US$ 3.6 billion should it have curbed COVID-19 disruptions on SRHR service provision (26). This finding suggests that morbidity and premature mortality due to COVID-19-related disruptions to SRHR services results in sizeable GDP losses in the Eastern and Southern Africa region. The associated economic losses can be averted if a cost-effective mix of interventions for SRHR services are made readily accessible to those who need them. This saving could be made through the full implementation of global strategy for women’s, children’s, and adolescent’s health (2016–2030) (27); the United Nations General Assembly Resolutions on the girl child (28) and the right to food (29); and the World Health Assembly Resolution on immunization (30) that underpin the framework to end preventable maternal and neonatal deaths. Moreover, the attainment of Sustainable development goals 3.1, 3.3, 3.7, 5.3, and 5.6 will require universal coverage of SRHR services including maternal health services, vaccines against preventable diseases, and integrated management of childhood diseases services during and after the COVID-19 pandemic. The monetary value of DALYs can also be used as evidence for investment case, for enhanced engagement and prioritization of SRHR interventions, especially focusing on areas that are greatly affected, such as Angola, South Africa, Democratic Republic of Congo, Zambia, Tanzania, and Kenya. Policymakers can also use these findings to advocate among the public and private sectors, for appropriate policies and regulations that promote access and continuity of essential services, whilst ensuring safety of beneficiaries.

A key strength of this study was the use of validated and standardized mortality data from authoritative national agencies, which allowed comparability across countries and time. However, the study had three limitations. First, it did not factor in home-based COVID-19 suspected deaths. This is more likely to lead to an overestimated DALYs due to COVID-19. Second, the human capital approach employed would value the DALYs accrued among the retired, unemployed and persons who cannot work due to severe disablement at zero, since they do not feature in total Gross domestic product (GDP) calculations. Therefore, it is inaccurate to assume that if all the causes of the DALYs loss were eradicated, then the Eastern and Southern Africa region would increase the total GDP by 100%. This is because a part of the population is either unemployed or above the compulsory retirement age, thereby excluding them from the labor force. Third, the human capital method estimates the value of health losses due to COVID-19 SRHR disruptions and does not provide estimates of the costs and consequences of alternative interventions.

## Conclusion

Analyzing both the health and economic impacts of COVID-19-associated SRHR service disruptions, highlights the trade-offs in deciding both where and how much is required to institute mitigation strategies. Evidence from this study can inform priority national strategic plans and advocate for sufficient investments to tackle SRHR issues, within the broader scope of health systems. Countries should strengthen their health systems functionality, incorporating and transforming lessons learned from shock events and avert health services disruption.

## Data availability statement

The original contributions presented in the study are included in the article/supplementary material, further inquiries can be directed to the corresponding author.

## Author contributions

HK, HC, and BD conceived the paper. HK, HC, SN, BD, DN, AA, AO, and JN-O undertook the development of the methodology and analytical approach. DM, WM, EO, EK, and AO conducted the data collection. HK, DM, WM, EO, EK, HC, SN, DN, JN-O, BD, AA, and AO conducted the data analysis and interpretation. DM, DN, WM, EO, and EK drafting of the manuscript. All authors critically reviewed it for important intellectual content, approved the version to be published, and agreed to be accountable for all aspects of the knowledge synthesis.

## Funding

This research was funded by the Swedish government, as part of the 2gther4SRHR project (Award 67353).

## Conflict of interest

The authors declare that the research was conducted in the absence of any commercial or financial relationships that could be construed as a potential conflict of interest.

## Publisher’s note

All claims expressed in this article are solely those of the authors and do not necessarily represent those of their affiliated organizations, or those of the publisher, the editors and the reviewers. Any product that may be evaluated in this article, or claim that may be made by its manufacturer, is not guaranteed or endorsed by the publisher.

## References

[1] UNDP. Articulating the pathways of the socio-economic impact of the coronavirus pandemic on the Kenyan economy. Policy Br. (2020) 4:33

[2] Mann DM Chen J Chunara R Testa PA Nov O. COVID-19 transforms health care through telemedicine: evidence from the field. J Am Med Informatics Assoc. (2020) 27:1132–5. doi: 10.1093/jamia/ocaa072, PMID: 32324855 PMC7188161

[3] Social stigma associated with COVID-19 – World | ReliefWeb. (2020). Available at: https://reliefweb.int/report/world/social-stigma-associated-covid-19 (Accessed May 22, 2020).

[4] Cabore JW Karamagi HC Kipruto HK Mungatu JK Asamani JA Droti B. COVID-19 in the 47 countries of the WHO African region: a modelling analysis of past trends and future patterns. Lancet Glob Heal. (2022) 10:e1099-14. doi: 10.1016/S2214-109X(22)00233-9, PMID: 35659911 PMC9159735

[5] Starrs AM Ezeh AC Barker G Basu A Bertrand JT Blum R. Accelerate progress—sexual and reproductive health and rights for all: report of the Guttmacher–Lancet Commission. Lancet. (2018) 391:2642–92. doi: 10.1016/S0140-6736(18)30293-9, PMID: 29753597

[6] Desa UN. United Nations Department of economic and social affairs. Popul Div World Popul Prospect. (2019)

[7] Implementation of the ESA commitment. (2022). Available at: https://www.giz.de/en/worldwide/35216.html (Accessed April 14, 2022).

[8] Hall KS Samari G Garbers S Casey SE Diallo DD Orcutt M. Centring sexual and reproductive health and justice in the global COVID-19 response. Lancet. (2020) 395:1175–7. doi: 10.1016/S0140-6736(20)30801-1, PMID: 32278371 PMC7146687

[9] Maula J. Impact of COVID-19 on gender equality and Women’s empowerment in east and Southern Africa. UN Women. (2021):1–62.

[10] Decker MR Wood SN Thiongo M Byrne ME Devoto B Morgan R. Gendered health, economic, social and safety impact of COVID-19 on adolescents and young adults in Nairobi, Kenya. PLoS One. (2021) 16:e0259583. doi: 10.1371/journal.pone.0259583, PMID: 34752473 PMC8577767

[11] Church K Gassner J Elliott M. Reproductive health under COVID-19–challenges of responding in a global crisis. Sex Reprod Heal Matters. (2020) 28:1773163. doi: 10.1080/26410397.2020.1773163, PMID: 32441213 PMC7887964

[12] UNFPA. Interim technical note impact of the COVID-19 pandemic on family planning and ending gender-based violence, female genital mutilation and child marriage. USA and Australia: Avenir Health, Johns Hopkins University and Victoria University.

[13] Impact of COVID-19 on sexual and reproductive health rights in Kenya – Hakijamii. (2022). Available at: https://www.hakijamii.com/?p=6268 (Accessed April 14, 2022).

[14] Eghtessadi R Mukandavire Z Mutenherwa F Cuadros D Musuka G. Safeguarding gains in the sexual and reproductive health and AIDS response amidst COVID-19: the role of African civil society. Int J Infect Dis. (2020) 100:286–91. doi: 10.1016/j.ijid.2020.08.086, PMID: 32920231 PMC7484728

[15] Sully E Biddlecom A Darroch JE Riley T Ashford LS Lince-Deroche N. Adding it up: investing in sexual and reproductive health 2019. New York: Guttmacher Institute. (2020).

[16] WHO, UNICEF, UNFPA. Module 1: understanding modelling approaches for sexual, reproductive, maternal, newborn, child and adolescent health, and nutrition. World Health Organization. (2021).

[17] Salje H Tran Kiem C Lefrancq N Courtejoie N Bosetti P Paireau J. Estimating the burden of SARS-CoV-2 in France. Science. (2020) 369:208–11. doi: 10.1126/science.abc3517, PMID: 32404476 PMC7223792

[18] LiST visualizer. (2022). Available at: https://listvisualizer.org/ (Accessed April 13, 2022).

[19] Moreno-Ternero JD Platz TT Østerdal LP. QALYs, DALYs, and HALYs: a unifying framework for the evaluation of population health. J Health Econ. (2023) 87:102714. doi: 10.1016/j.jhealeco.2022.102714, PMID: 36516569

[20] Viscusi WK Aldy JE. The value of a statistical life: a critical review of market estimates throughout the world. J Risk Uncertain. (2003) 27:5–76. doi: 10.1023/A:1025598106257

[21] World Bank. World development indicators 2017. World Dev Indic. (2017). Available at: https://openknowledge.worldbank.org/handle/10986/26447 (Accessed April 13, 2022).

[22] Viscusi WK Masterman CJ. Income elasticities and global values of a statistical life. J Benefit Cost Anal. (2017) 8:226–50. doi: 10.1017/bca.2017.12

[23] Soma-Pillay P Moodley J Pattinson R Fawcus S Gebhardt S Niit R. The effect of the first wave of COVID-19 on use of maternal and reproductive health services and maternal deaths in South Africa. Obstetr Gynaecol Forum. (2020) 30:38–46.

[24] Chattu VK Lopes CA Javed S Yaya S. Fulfilling the promise of digital health interventions (DHI) to promote women’s sexual, reproductive and mental health in the aftermath of COVID-19. Reprod Health. (2021) 18:1–8. doi: 10.1186/s12978-021-01168-x 34088319 PMC8177268

[25] Islam N Jdanov DA Shkolnikov VM Khunti K Kawachi I White M. Effects of covid-19 pandemic on life expectancy and premature mortality in 2020: time series analysis in 37 countries. bmj. (2021) 375. 10.1136/bmj-2021-066768 PMC8564739 34732390

[26] Kirigia JM Mwabu GM. The monetary value of disability-adjusted-life-years lost in the East African community in 2015. Modern Economy. (2018) 9:1360–77. doi: 10.4236/me.2018.97087

[27] WHO. Every woman every child 2015 the global strategy for women’s, children’s and adolescents health (2016-2030). World Health Organization. (2015). 10.2471/BLT.16.174714 PMC4850547 27147756

[28] UN. The girl child: resolution / adopted by the general assembly. (2017). Available at: https://digitallibrary.un.org/record/1469997?ln=en (Accessed April 14, 2022).

[29] UN. The right to food: resolution / adopted by the general assembly. (2018). Available at: https://digitallibrary.un.org/record/1467246?ln=en (Accessed April 14, 2022).

[30] World Health Assembly, 70. Strengthening immunization to achieve the goals of the global vaccine action plan. Geneva: World Health Organizations (2017).

